# Mind over Matter: Testing the Efficacy of an Online Randomized Controlled Trial to Reduce Distraction from Smartphone Use

**DOI:** 10.3390/ijerph17134842

**Published:** 2020-07-05

**Authors:** Melina A. Throuvala, Mark D. Griffiths, Mike Rennoldson, Daria J. Kuss

**Affiliations:** 1 International Gaming Research Unit, Psychology Department, Nottingham Trent University, Nottingham NG1 4FQ, UK; mark.griffiths@ntu.ac.uk (M.D.G.); daria.kuss@ntu.ac.uk (D.J.K.); 2 Psychology Department, Nottingham Trent University, Nottingham NG1 4FQ, UK; mike.rennoldson@ntu.ac.uk

**Keywords:** distraction, smartphones, social media, intervention, randomized controlled trial, social media addiction

## Abstract

Evidence suggests a growing call for the prevention of excessive smartphone and social media use and the ensuing distraction that arises affecting academic achievement and productivity. A ten-day online randomized controlled trial with the use of smartphone apps, engaging participants in mindfulness exercises, self-monitoring and mood tracking, was implemented amongst UK university students (*n* = 143). Participants were asked to complete online pre- and post-intervention assessments. Results indicated high effect sizes in reduction of smartphone distraction and improvement scores on a number of self-reported secondary psychological outcomes. The intervention was not effective in reducing habitual behaviours, nomophobia, or time spent on social media. Mediation analyses demonstrated that: (i) emotional self-awareness but not mindful attention mediated the relationship between intervention effects and smartphone distraction, and (ii) online vigilance mediated the relationship between smartphone distraction and problematic social media use. The present study provides preliminary evidence of the efficacy of an intervention for decreased smartphone distraction and highlights psychological processes involved in this emergent phenomenon in the smartphone literature. Online interventions may serve as complementary strategies to reduce distraction levels and promote insight into online engagement. More research is required to elucidate the mechanisms of digital distraction and assess its implications in problematic use.

## 1. Introduction

Attentional focus is one of the most fundamental resources and a key to successful and high-order work [1]. In the attention economy [2], multiple online and offline activities compete for an alternative share of attention [3]. This trend is expected to grow in the face of increasing communication complexity and information overload [4], which is becoming even more prevalent partially due to the vast online accessibility, immediacy and convenience of smartphones, acting as a major motivational pull for engagement [5] and prompting constant multitasking and frequent attentional loss [6]. There are currently more than 3.5 billion smartphone users [7] and smartphone use is an emergent area of research [8,9,10]. Emerging evidence on cognitive function has shown that smartphone availability and daily interruptions compete with higher-level cognitive processes creating a cognitive interference effect [11,12,13,14,15], associated with poorer cognitive functioning [16,17,18,19], performance impairments in daily life [20] and potential supplanting of analytical thinking skills by “offloading thinking to the device” [21] (p. 473). In spite of such initial evidence, there are cognitive correlates within the smartphone context, such as distraction, which have been less explored in the literature. Studies report that students use their smartphones for more than 25% of effective class duration, and smartphone distractions occur every 3–4 min, for over a minute in duration [22]. Student focus on any single task is reported to last 3–5 min [23] with excessive smartphone use hindering academic performance as a result of allowing goal-irrelevant information to compete with goal-relevant tasks [24,25]. Therefore, examining the processes involved in the occurrence of distraction as well as protective strategies for its containment is timely. The present study evaluates the efficacy of evidence-based mediating strategies in reducing distraction employed in an online randomized controlled trial.

Distraction is an emotion regulation coping strategy used to deflect attention from the task at hand in order to relieve emotional distress, reflected as difficulty in concentrating and maintaining goal-focused behaviour, with an adaptive function in negative affect situations [26,27,28,29,30]. Smartphone distraction constitutes an emergent concern, operationally defined as the disruption in attention due to: (i) external cues received (i.e., notifications), (ii) cognitive salience (i.e., internal cues) of the smartphone and social media, or (iii) cognitive avoidance (i.e., coping mechanism) for emotion regulation [17,31,32,33]. Checking behaviours, frequently engaged in during smartphone use, are associated with repeated external or internal interruptions, leading to attentional micro-disengagements and distraction [20,31,34]. According to the control model of social media engagement [5], this may occur as need to control online content, relationships and self-presentation produces an attentional conflict (offline vs. online or platform/activity switch), arousal and distraction, leading either to facilitation (by the presence of online others) [35,36] and heightened engagement or shallow processing, when engaged in parallel cognitively demanding tasks. Therefore, constant disruptions may cause a rise in attention problems and hyperactivity levels [37] as a result of allowing goal-irrelevant information to compete with goal-relevant tasks [24,25] with impacts on wellbeing, productivity and academic achievement, particularly amongst young people [22,38,39,40,41]. A large contributor to this effect is excessive social media use, which has been suggested as a vulnerability factor for problematic smartphone use [42,43,44]. To date, the effects of smartphone use on student outcomes may still be small [45].

### 1.1. Distraction and Its Relation to Other Psychological Constructs in the Smartphone Literature

*Online vigilance*. Distraction by smartphone use appears to be activated by internal thoughts or external cues interfering with other tasks, which may be driven by online vigilance—a constant preoccupation with online content, leading to salience, monitoring and prompting urges to check [46], resulting in strong habitual behaviour [47,48]. Salience of online content has been found to be negatively associated with affective wellbeing and life satisfaction, particularly when thoughts are negative [49].

*Attention impulsiveness and habitual smartphone use*. Attention impulsiveness has also been implicated in smartphone distraction, reinforced by rewarding, habitual checking behaviours [47], and has a significant relationship with problematic smartphone use [50]. Recent evidence also suggests symptom severity of problematic social media use to be mainly associated with attention impulsiveness and difficulties with inhibitory control or executive control functions [51], task performance [52] and chronic media multitasking [25]. This is intensified in a low interest academic context, reducing lecture comprehension [53], level of motivation, and fluid intelligence [54].

*Fear of Missing Out (FoMO) and Nomophobia (NoMO)*. FoMO—the fear of being excluded from rewarding social experiences – and NoMO – the fear of no access to a mobile device—have both been evidenced in the smartphone literature as triggering a need to be in constant contact and reinforcing use [55,56,57,58,59,60,61,62]. Therefore, FoMO could be a main driver of distraction due to the propensity to be present in the positive experiences others are having, depicted in online content. FoMO has been associated with depression, smartphone addiction, anxiety, mindfulness and wellbeing [63], negative affectivity, problematic smartphone use, and levels of online social engagement [60]. 

*Stress, anxiety, emotion regulation and problematic use*. Socio-emotional correlates of FoMO have included negative affect, rejection sensitivity, and high stress levels [64], and reviews have suggested a small-to-medium association between smartphone use and stress and anxiety [65]. Therefore, negative emotional states may be a precursor to smartphone distraction and its use may be motivated by emotion regulation. Relief of negative emotions and psychological states along with emotional gains from smartphone use have been found to be significantly higher for Generation Z (individuals born between 1995 and 2015) [66] and could be an outcome of difficulties with emotion regulation, creating a vicious cycle sustaining overreliance for coping [67] and dysfunctional metacognitive beliefs among problematic users [68]. Smartphone unavailability and intolerance of uncertainty have been evidenced in problematic smartphone use [69,70], and affect perceived stress and mental wellbeing [71]. Concerns for the emotional and behavioural consequences of excessive smartphone and social media use have been addressed [9,72,73,74,75]. However, what constitutes problematic online behaviour needs constant conceptual and methodological re-evaluation [76] as engagement with new products/platforms emerges.

*Mindfulness, self-monitoring and mood tracking*. Self-monitoring of social media activity, self-exclusion from specific platforms, and the practice of mindfulness are considered successful wellbeing practices [77,78]. Mindfulness, defined as the purposeful, non-judgemental awareness of the presenting experience [79], facilitates the sustaining of on-task behaviours [80], affecting attention, affect regulation, body awareness, and self-perception [81,82,83], and has been used in gambling harm-reduction and substance use disorders, with intervention effects reducing cravings, post-traumatic symptoms, and negative affect [84,85,86,87,88,89,90]. Mindfulness has been negatively associated with distraction, suggesting that one’s awareness of own thought wandering (meta-awareness) may decrease the frequency of distraction [17] and aid academic attainment [91]. Self-monitoring of mood (also defined as mood tracking) has been found to boost overall emotional self-awareness [92], which can in turn lead to improvements in emotional self-regulation [93]. Therefore, these strategies could be trialled to help diminish attentional bias occurring within the context of social media and smartphone use [94,95].

### 1.2. Smartphone Mental Health Apps (MHapps) and Online Randomized Controlled Trials

Digital wellbeing apps or MHapps (apps that track an individual’s behaviour, i.e., time spent online, or that aid cognitive, emotional and/or behavioural wellbeing) [96] have been suggested as supporting self-awareness and self-regulation [97] and utilized in mental healthcare given their functionality, accessibility, higher adherence rates, real-time assessment, low-cost and for their intervention potential [98,99]. The literature suggests that evidence-based apps may be efficacious in raising self-awareness, mental health literacy and wellbeing, self-efficacy, and ability to cope [96,100,101,102]. Online psychological interventions are becoming more prominent in the digital age [103], rendering numerous positive health outcomes [102,104,105,106,107,108], complementing service provision and recognized by governmental health institutions (e.g., National Institute for Health and Care Excellence (NICE) in the UK) [109]. However, more research is required to determine the comparative effectiveness of these therapies and their components [110] in improving mental health and wellbeing and rigorous objective evaluation beyond their developers is required. 

To date, there have been a small number of internet-based interventions associated with device use in university settings. Distraction is not considered a dysfunctional construct by itself, but has been implicated in emotion regulation, ADHD, and other disorders [111,112,113], and has been minimally examined in the context of the digital environment with no evidence to date as to strategies that could ameliorate its occurrence [114]. Therefore, the aim of the present study was to test the preliminary efficacy of an online intervention based on cognitive behavioural principles (i.e., self-monitoring, mood tracking, and mindfulness) to reduce distraction and related psychological outcomes (i.e., stress) among university students. Given: (i) young adults are keen users of smartphone apps, with increased vulnerability to self-regulation and technology use [74], (ii) the high stakes for academic achievement, and (iii) the similarity in processes observed between gambling addiction and social media overuse [115], the strategies of *mindfulness, activity monitoring*, and *mood tracking* utilized in gambling harm-reduction [86,116,117] are employed in the present study. These strategies were delivered and facilitated through the use of smartphone MHapps and were tested for their efficacy in reducing levels of distraction and related psychological outcomes and their role in inducing changes in wellbeing [118,119,120]. The following hypotheses were formulated:

**Hypothesis 1** **(H1).** *Compared to the control condition at follow-up, students receiving the intervention would report: (i) lower rates of smartphone distraction, smartphone and social media use duration, impulsivity, stress, problematic social media use, FoMO and NoMO and (ii) higher levels of mindful attention, emotional self-awareness, and self-efficacy.*

**Hypothesis 2** **(H2).** *At follow-up, high distractors (HDs) compared to low distractors (LDs) (based on a median-split analysis) would show a greater reduction in distraction and significant improvement in outcomes.*

**Hypothesis 3** **(H3).** *The intervention will mediate the relationship between (i) mindful attention and smartphone distraction, and (ii) emotional awareness and smartphone distraction. Additionally, online vigilance will mediate the relationship between smartphone distraction and problematic social media use.*

To the authors’ knowledge and given the novelty of the construct of smartphone distraction, this is the first study to examine a preliminary online randomized controlled trial via MHapps for the reduction of smartphone distraction. The present study fills a gap in the smartphone literature by assessing the efficacy of engaging with behaviour change strategies (i.e., mindfulness, self-monitoring, and mood-tracking) used successfully in gambling harm prevention for the reduction of distraction. 

## 2. Materials and Methods 

### 2.1. Design

The present study tested the efficacy of a ten-day online app-delivered randomized controlled trial (RCT) based on cognitive-behavioural principles to reduce distraction (primary outcome) and a number of secondary psychological outcomes: self-awareness, mindful attention, FoMO, anxiety, and depression among university students. RCTs are considered the gold standard in intervention effectiveness despite limitations addressed by scholars [121,122], primarily for the lack of external validity or methodological choices [123]. A pragmatic psychosocial intervention with an RCT design was chosen [124]. The duration of the intervention was set given a pragmatic consideration of the free use period of one of the apps (*Headspace*) and, secondly, due to the preliminary nature of this investigation. Consolidated Standards of Reporting Trials (CONSORT) guidelines were followed in the protocol and the procedures and reporting of the intervention [125].

The intervention involved the active engagement for the period of ten consecutive days with three smartphone apps serving three different functions: to assess smartphone and social media use, conduct mindfulness sessions with an emphasis on eliminating distraction, and track mood and assess its impact on distraction, stress, self-regulation, and other measures. Interaction with apps was encouraged to: (i) raise emotional awareness of common mood states, such as feeling down, worried, or stressed through mindfulness, (ii) guide basic smartphone monitoring, focusing skills, and awareness, and (iii) provide insight through mood tracking (Table 1). To further support active engagement with these intervention components, eligible participants were asked to keep a daily online activity log for the duration of the intervention (i.e., the number of screen-unlocks and the time of day and number of minutes for which the smartphone was used, usefulness of apps, etc.), to aid time perception of daily activities, raise awareness levels, and help increase the accuracy of self-reporting and adherence to the intervention [126,127]. Promoting self-awareness of media use and understanding of own behaviour was a key target of the intervention in order to curb distraction. The study was reviewed and approved (No. 2018/226) by the research team’s university ethics committee.

### 2.2. Participants

Participants were recruited using convenience and snowball sampling techniques. After gaining institutional ethical approval, the study was advertised to students through the research credit scheme, in university lectures and labs, and to the public through social media as an online intervention to assess the reduction of smartphone distraction. This experimental intervention demanded a significant time involvement and offering incentives increased the chances of participation and completion of the full ten-day intervention. In return for participation, students were offered either research credits or entry in a prize draw (£50 gift cards). Participants were included in the study based on two screening criteria: regular smartphone and social media usage. Only those affirming both and granting consent were able to continue with participation. Following the completion of the survey, participants were allocated to one of the two conditions (intervention [IG] or control [CG]) and further instructions for participation in the intervention were provided depending on the allocation condition. After initially providing age and gender demographics, participants responded to survey items regarding habitual smartphone and social media behaviour (estimates of duration of use), smartphone distraction severity, trait self-regulation, trait mindfulness and other psychological constructs (detailed in “Materials”). The survey took approximately 25 min to complete. 

**Figure 1:**
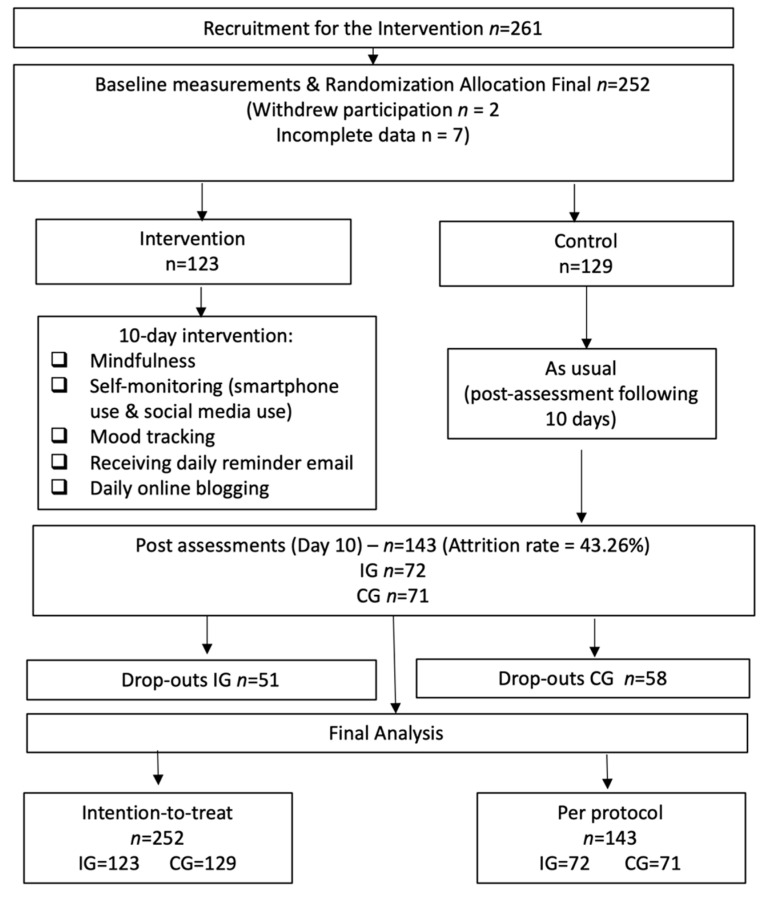
Participant flow in the intervention.

A total of 261 participants were recruited who participated in the baseline assessment. Of these, 155 were undergraduate Psychology students in the UK (59.3%). The sample comprised 47 males (18%) and 214 females (82%), with an age range of 18 to 32 years (*M* = 20.72, *SD* = 3.12). Figure 1 depicts the flow of participants through the study procedures. After the baseline assessment, during the intervention period two individuals of the intervention group withdrew from the study and were not considered in the analysis. From the 259 remaining participants, seven were removed due to providing 90% incomplete data. The final sample considered at baseline was 252 participants (intention to treat (ITT) group) and included 123 participants in the intervention group and 129 in the control group. Participants who completed both assessments were considered in the per-protocol analysis (PP) (*n* = 143, 56% of the original sample), with 72 participants comprising the IG and 71 participants the CG. 

### 2.3. Materials 

The survey consisted of sociodemographic and usage data (questions related specifically to smartphone and social media use [hours per day]). The demographic questions and user-related questions had open responses (i.e., “How many hours per day do you use social media?”). The following scales were used for the psychological measures of the study: 

The Smartphone Distraction Scale [138] is a newly developed scale comprising of 16 Likert-type items. The scale comprises four factors: attention impulsiveness, online vigilance, emotion regulation, and multitasking. Scores range from 1 (almost never) to 5 (almost always) with higher scores representing a greater degree of distraction. Individual items on the test were summed to give composite scores. Sample items included in the scale are the following: “I get distracted by my phone notifications”, and “I constantly check my phone to see who liked my recent post while doing important tasks”. The scale has demonstrated good psychometric properties [138] and excellent reliability in the present study with a Cronbach’s alpha of 0.90 for Time 1 (T1) and 0.88 for Time 2 (T2). 

The Mindful Attention Awareness Scale (MAAS) [139] is a 15-item assessment tool that assesses the dispositional tendency of participants to be mindful in everyday life and has been validated among young people, university students and community samples [139,140]. Item statements reflect experience of mindfulness, mindlessness in general and specific daily situations and are distributed across a range of cognitive, emotional, physical, interpersonal, and general domains. Response options are based on a six-point Likert scale from 1 (almost always) to 6 (almost never). Scores were averaged across the 15 items to obtain an overall mindfulness score with higher scores reflecting higher levels of dispositional mindfulness. Sample items include “I could be experiencing some emotion and not be aware of it until sometime later” and “I find it difficult to stay focused on what’s happening in the present” and exhibited a high degree of internal consistency in the present study with a Cronbach’s alpha of 0.92 for T1 and 0.93 for T2.

The Emotional Self-Awareness Scale (ESAS) [92] was used to assess ESA and comprises five variables: recognition, identification, communication, contextualization, and decision making. The scale consists of 32 items (e.g., “I usually know why I feel the way I do”) rated from 0 (strongly disagree) to 4 (strongly agree). The total ESA score ranged from 0 to 128, and sub-scale items are combined to produce a composite score with higher scores indicating higher ESA. The ESAS has presented reasonable internal consistency (Cronbach’s alpha = 0.72, 0.69, and 0.76 for pre-test, post-test and six-week follow-up) [92]. The scale has demonstrated good validity in prior studies [92,101] and adequate internal consistency in the present study (Cronbach’s alpha of 0.87 for T1 and 0.86 for T2).

The Perceived Stress Scale (PSS) [141] is one of the most widely used scales to assess perceived stress and the degree of unpredictability, uncontrollability, and burden in various situations. The scale used was the 10-item version rated from 0 (never) to 4 (very often) with sample items such as “In the last month, how often have you felt that you were unable to control the important things in your life?”, and “In the last month, how often have you felt that you were on top of things?” Scores are obtained by summing the items, with the higher score indicating more perceived stress. The scale possesses good psychometric properties [142] and its internal consistency in the present study was 0.86 for T1 and 0.83 for T2.

The seven-item Generalized Anxiety Disorder Scale (GAD-7) [143] is a brief clinical measure that assesses for the presence and severity of Generalized Anxiety Disorder (GAD). The self-report scale asks how often during the last two weeks individuals experienced symptoms of GAD. Total scores range from 0–21 with cut-off scores of 5, 10, and 15 being indicative of mild, moderate, and severe anxiety, respectively. Increasing scores on the GAD-7 are strongly associated with greater functional impairment in real-world settings. Sample items are rated from 0 (not at all) to 3 (nearly every day) and sample items include: “Feeling nervous, anxious or on edge” and “Trouble relaxing”. The scale has been widely used and considered a valid and reliable screening tool in previous research, presenting good reliability, factorial and concurrent validity [144,145], and demonstrated excellent internal consistency in the present study (α = 0.93 Τ1 and α = 0.90 for T2).

The Self-Report Behavioural Automaticity Index (SRBAI) [146] was used to assess habitual strength. The four-item scale was used to assess the degree of automaticity and contained items such as: “Using social media on my smartphone is something…I do automatically” and “I start doing before I realize I’m doing it”. Participants indicate their agreement with each item on a Likert scale ranging from 1 (does not apply at all) to 7 (fully applies). Scores were averaged across items to obtain an overall habit score, with higher scores indicating stronger habitual smartphone use behaviour. The scale has been reported as psychometrically sound in previous studies with good reliability, convergent and predictive validity [146,147] and demonstrated good internal consistency in the present study with a Cronbach’s alpha of 0.87 (T1) and 0.89 (T2). 

The Generalized Self-Efficacy Scale (GSE) [148] is a widely used psychometric instrument comprising ten items that assess perceived self-efficacy (“I can always manage to solve difficult problems if I try hard enough.”). Items are rated on a four-point scale ranging from 1 (not at all true) to 4 (exactly true). The GSE has demonstrated satisfactory internal consistency and validity across studies [149,150]. Cronbach’s alpha in the present study was 0.90 (T1) and 0.88 (T2).

The Online Vigilance Scale (OVS) [46] is a 12-item Likert scale which assesses a relatively new construct in the internet-related literature, referring to individuals’ cognitive orientation towards online content, expressed as cognitive salience, reactivity to online cues and active monitoring of online activity. Sample items include “My thoughts often drift to online content” and “I constantly monitor what is happening online”. Scale items are rated on a four-point Likert scale from 1 (does not apply at all) to 4 (fully applies). Higher mean scores indicate a higher degree of online vigilance. The scale has evidenced sound construct and nomological validity and high internal consistency [46,49,78]. The Cronbach’s alpha in the present study was 0.89 (T1) and 0.87 (T2).

The eight-item Barratt Impulsiveness Scale-Alternative Version (BIS-8) [151] is a psychometrically improved abbreviated version of the 11-item BIS scale [151] presenting good construct and concurrent validity in young populations [152,153]. The scale assesses impulsive behaviour and poor self-inhibition and uses a four-point Likert scale from 1 (do not agree) to 4 (agree very much). Sample items include: “I do things without thinking” and “I act on the spur of the moment”. Cronbach’s alpha coefficient in the present study was 0.85 (T1) and 0.86 (T2). 

The Deficient Self-Regulation Measure [154] is a seven-item scale assessing deficient self-regulation in videogame playing adapted for unregulated internet use [155]. The scale is rated on a seven-point Likert scale from 1 (almost never) to 7 (almost always) and has demonstrated sound psychometric properties [154]. The scale was adapted for smartphone use with sample items such as “I would go out of my way to satisfy my urges to use social media” and “I have to keep using social media more and more to get my thrill”. The original scale and its adaptation has presented satisfactory psychometric properties [154,155]. The Cronbach’s alpha coefficient in the present study was 0.89 (T1) and 0.87 (T2). 

The Bergen Social Media Addiction Scale (BSMAS) [115,156,157,158] is a six-item self-report scale for assessing social media addiction severity based on the framework of the components model of addiction (salience, mood modification, tolerance, withdrawal, conflict, and relapse) [159]. Each item examines the experience of using social media over the past year and is rated on a five-point Likert scale from 1 (very rarely) to 5 (very often), producing a composite score ranging from 6 to 30. Higher BSMAS scores indicate greater risk of social media addiction severity. A sample question from the BSMAS is “How often during the last year have you used social media so much that it has had a negative impact on your job/studies?” A cut-off score over 19 indicates problematic social media use [160]. The BSMAS has presented sound psychometric properties [115,156,157,158] with high internal consistency (α = 0.82) [161]. The Cronbach’s alpha in the present study was 0.91 (T1) and 0.87 (T2).

The Fear of Missing Out Scale (FoMOS) [162] includes ten items and asks participants to evaluate the extent to which they experience symptoms of FoMO. The scale is rated on a seven-point Likert scale from 1 (not at all true) to 5 (extremely true of me). The statements include: “I fear others have more rewarding experiences than me... I get anxious when I don’t know what my friends are up to...It bothers me when I miss an opportunity to meet up with friends...”. A total score was calculated by averaging the scores, with higher mean scores indicating a greater level of FoMO. This instrument has demonstrated good construct validity [162,163], and good internal consistency with Cronbach’s alphas of α = 0.93 [164] and 0.87 [64] with α = 0.87 in the present study. 

The Nomophobia Questionnaire (NMP-Q) [165] comprises 20 items rated using a seven-point Likert scale from 1 (strongly disagree) to 7 (strongly agree). Total scores are calculated by summing up responses to each item, resulting in a nomophobia score ranging from 20 to 140, with higher scores corresponding to greater nomophobia severity. NMP-Q scores are interpreted in the following way: 20 = absence of nomophobia; 21–59 = mild level of nomophobia; 60–99 = moderate level of nomophobia; and 100+ = severe nomophobia. The scale has demonstrated good psychometric properties [165,166] with Cronbach’s alphas of 0.94 [165] and 0.95 [167]. In the present study, internal consistency was: 0.89 for (T1) and 0.88 for (T2) respectively.

### 2.4. The Intervention

The intervention initially involved the search and identification of appropriate mobile apps (in both the Apple iTunes store and the Android Google Play store) for daily self-monitoring of social media activity for mindfulness practices and mood tracking. The apps needed to be freely available in order to be accessible by the participants. Due to time limitations, the development of an app that would encompass all three features (mindfulness of distraction, self-monitoring, and mood-tracking) was deemed adequate for the study given the ample availability of well-designed products offering these services. The following three freely available smartphone lifestyle apps were utilized: (i) Antisocial (screen time): to self-monitor screen time/social media use and for voluntary self-exclusion (block app after time limit is reached), (ii) Headspace (mindfulness): brief mindfulness sessions, (iii) Pacifica (mood tracking): the app encouraged monitoring and tracking an individual’s emotional state at various times during the day to enhance awareness. 

At the outset of the study, participants were directed to an information statement followed by the digital provision of informed consent before responding to the questions. At the end of the survey, they were automatically assigned through the automatic randomization procedure used by the online survey platform *Qualtrics* to either an intervention or a control group. Therefore, the intervention was double-blind (to participants and investigators). Participants assigned to the *IG* were asked to download the apps onto their smartphones and to actively engage with all three apps daily for 10 days, which was the maximum free period offered by one of these apps. Participants were encouraged to engage with mindfulness/focusing exercises to track their emotional state during the day and monitor patterns in their wellbeing as well as report daily on smartphone usage rates. Thereafter, participants received daily notifications via email for the duration of the intervention to remind them to provide online reports about their own social media usage rates, apps accessed, checking frequency, potential self-restriction from use, and satisfaction with the intervention. This process was used to motivate engagement with the apps and accountability. Efficacy was evaluated by having a CG condition where participants did not engage in any app use and only completed assessments on the first and tenth day. The target of the intervention was to induce a more mindful state, raise awareness of media and smartphone use, enhance self-regulation and therefore reduce distractions and time spent on smartphones and indirectly on social media by using these apps.

### 2.5. Data Analysis 

#### 2.5.1. Sample Size Estimation 

The sample size for the RCT was determined a priori using G*Power v.3 software for the expected increased effectiveness of the intervention compared to control on the primary outcome distraction at post-assessment (T2). Empirical reviews [168] have suggested a median standardised target effect size of 0.30 (interquartile range: 0.20–0.38), with the median standardised observed effect size 0.11 (IQR 0.05–0.29). The present study was a low-threshold intervention for a non-clinical population, so a mean effect of *d* = 0.30 was expected. With a power of 1-ß = 0.8, and a significance level of α = 0.05, the sample size was calculated to be *n* = 95 participants per group to find between- and within-group effects. To account for attrition rates in online interventions and control for both Type I and II error rates, *n* = 125 participants per group were targeted for recruitment [169]. 

#### 2.5.2. Data Cleaning, Assumption Testing and Descriptive Analysis

All data were analysed through SPSS v.25 (Chicago, IL, USA). Preliminary data analyses included examining the data for data entry errors, normality testing, outliers, and missing data. Seven cases were treated with listwise deletion due to a very high percentage of incomplete data at baseline, resulting in a final sample size of 252. For the rest of the dataset, Little’s Missing Completely at Random (MCAR) test showed that data were missing completely at random (*p* = 0.449). Multiple imputation was used to complete the dataset for the baseline analysis and for the non-completers from post-intervention assessment based on patterns of missingness. The data were also checked to ensure that all assumptions for the outlined statistical analyses were satisfied. The Kolmogorov-Smirnov test was used to evaluate the normal distribution of the variables, and skewness and kurtosis values were examined. For both assessments, all self-report data were normally distributed. Assumptions of *t*-tests included normality, homogeneity of variance, and independence of observations. Violations of the assumption of homogeneity of variance were tested using Levene’s test of equality of variances [170]. Descriptive statistics were conducted to summarize the demographic characteristics of the sample as well as scores for the self-reported and performance-based measures of interest (i.e., stress). Pearson’s correlations examined bivariate relationships between smartphone distraction and psychological variables, and frequency of smartphone and social media use (presented in Table 3). 

#### 2.5.3. Randomization and Risk of Bias

While allocation randomisation aimed to reduce any differences between the groups at baseline, a series of independent sample *t*-tests for the continuous variables and chi-square tests for the categorical variables (gender, ethnicity and education and relationship status) were conducted to analyse group mean differences and compare the baseline and post-intervention outcomes for the control and intervention groups. These were also applied at post-intervention outcomes for both the control and the intervention group. A decrease from the baseline to the post-intervention assessment was hypothesised for the primary outcomes of smartphone distraction, stress, anxiety, deficient self-regulation, FoMO and NoMO and an increase was hypothesized for mindful attention, self-awareness and self-efficacy. 

Following the descriptive analysis, data from the baseline and post-intervention assessments were analysed to test each of the hypotheses provided to inform the assessment of the intervention efficacy. Two approaches to analysis were adopted. First, to isolate any effect of the intervention, a per-protocol (PP) analysis was conducted to maintain the baseline equivalence of the intervention group produced by random allocation [171]. However, given the limitations to this first analysis approach and to minimise biases resulting from noncompliance, non-adherence, attrition or withdrawal [172,173], analysis was performed also on an intention-to-treat (ITT) basis [172]. However, these results were not reported in the present study. 

#### 2.5.4. Analysis of Intervention Effects and Testing of Hypothesized Mechanisms

The effects of the intervention were assessed with an analysis of covariance (ANCOVA), with a minimum significance level at *p* < 0.05. ANCOVA was chosen given that it is quite robust with regard to violations of normality, with minimal effects on significance or power [174,175] with any differences between the groups at baseline, for the various assessments being used as covariates in the model and considered artefacts of the randomisation [176]. Co-varying for baseline scores supported the analysis in two ways. First, while randomisation aimed to reduce any pre-intervention differences between the groups, residual random differences may have occurred. Accounting for such differences isolated the effect of the intervention. Partial eta-squared were used as measures of strength of association [177]. To better understand the effect size of the intervention, it has been recommended to use the differences in adjusted means (standardized mean difference effect sizes) between the two groups, as standardising can easily distort judgements of the magnitude of an effect (due to changes to the sample SD but not the population SD, which may bias the estimate of the effect size measure, such as Cohen’s *d*) [178]. As Cohen’s *d* has been reported in other RCT and pre-post intervention studies, Cohen’s *d* was estimated [179]. Finally, because the sample sizes of the two groups were unequal, Type III Sums of Squares were used for the ANCOVA. 

To test the third hypothesis and the hypothesized psychological mechanisms underlying the intervention results, three different mediation analyses were performed across the chosen psychological constructs using SPSS Statistics (version 25) and PROCESS (Model 4; [180,181,182,183]), using a non-parametric resampling method bootstrap with 5000 bootstrapped samples and bias-corrected 95% confidence intervals, to probe conditional indirect effects for the variables examined. These analyses were performed on the ITT sample in post-intervention results.

## 3. Results

### 3.1. Baseline Equivalence Evaluation

The *t*-test results for the pre-test scores found no significant differences between the groups, indicating independence. The post-test scores were significantly lower in the intervention group. For the smartphone distraction scale, the mean pre-test score was 58.06 (*SD* = 7.69) for the intervention group and 59.72 (*SD* = 8.08) for the control group. The mean post-test score was 39.70 (*SD* = 17.67) for the intervention and 58.78 (*SD* = 17.47) for the control group, respectively. The pre-test score mean was not significantly different between groups (*t* = −0.70, *ns*), but the post-test score mean was significantly lower for the intervention group than for the comparison group (*t* = −6.69, *p* < 0.001). The pattern was similar in the results for the other variables except for NoMO, habitual behaviour, and social media use per day. Table 2 provides a summary of the baseline *t*-test and chi-square outcomes and internal consistency for each scale at each measurement period. All scales demonstrated good internal consistency for the sample considered.

A series of Bivariate Pearson’s *r* correlation analyses was conducted to examine the results obtained amongst SDs and the secondary outcomes (Table 3). Smartphone distraction correlated significantly with problematic social media use (*r*(252) = 0.63, *p* < 0.01), anxiety (*r* (252) = 0.46, *p* < 0.01), online vigilance (*r* (252) = 0.51, *p* < 0.01), automaticity (*r* (252) = 0.57, *p* < 0.01), impulsivity (*r*(252) = 0.45, *p* < 0.01), deficient self-regulation (*r*(252) = 0.33, *p* < 0.01), smartphone use/day (*r*(252) = 0.31, *p* < 0.01), *p* < 0.01), FoMO (*r*(252) = 0.28, *p* < 0.01) and NoMO (*r*(252) = 0.51, *p* < 0.01). However, smartphone distraction correlated negatively with two variables: mindful attention (*r*(252) = −0.52, *p* < 0.01) and self-awareness (*r*(252) = −0.34, *p* < 0.01).

### 3.2. Intervention Efficacy Evaluation

**Figure 2:**
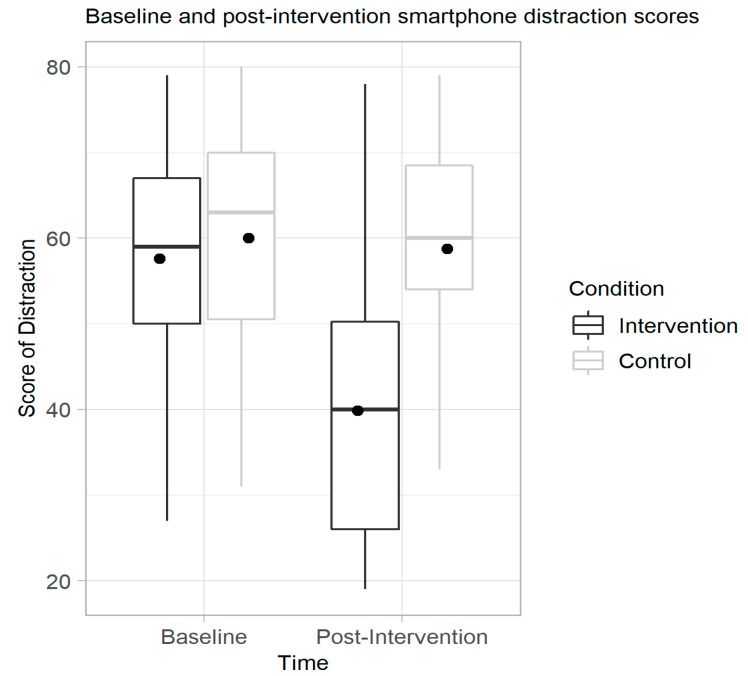
Per protocol smartphone distraction outcomes before and after the intervention.

To test H1 and assess the effect of the intervention on smartphone distraction, two separate ANCOVAs were conducted. First, to isolate any effect of the intervention, a per-protocol analysis was conducted. As depicted in Table 4, distraction outcomes decreased significantly for the intervention group from the baseline (intervention: *M* = 58.06, *SD* = 7.69; control: *M* = 59.72, *SD* = 8.08) to the post-intervention assessment (intervention: *M* = 39.70, *SD* = 17.67; control: *M* = 58.78, *SD* = 17.47), with a non-significant difference for the control group. As confirmed by Levene’s test, the outcome variances were homogenous. Confirming the homogeneity of the regression slopes, the interaction between the baseline scores and the experimental group was significant. There was a main effect of the intervention group on post-intervention distraction scores after controlling for baseline outcomes (*F*(1, 140) = 46.59, *p* < 0.001, *ηp^2^* = 0.250). The baseline scores were not a significant predictor of post-intervention values (*F*(1, 140) = 18.62, *p* = 0.117). Post-hoc tests indicated there was a statistically significant adjusted mean difference (*M* = −18.95, *SD* = 2.77, (*p* < 0.001) in reduction between IG compared to CG (Figure 2). For the ITT analysis, a main effect on the intervention group on post-intervention SDS outcomes after controlling for the baseline values was found (*F*(1, 250) = 96.88, *p* < 0.001, *ηp^2^* = 0.28). As indicated in Figure 2, post-hoc tests indicated there was a significant difference between IG and CG (*p* < 0.001). Comparing the estimated marginal means showed that there was an adjusted mean difference in reduction between IG (*M* = 39.56) compared to CG (*M* = 58.93). Consequently, across both analyses, this hypothesis was supported.

ANCOVA analyses for the secondary outcomes were also tested across both PP and ITT samples. Specifically, for the PP sample, main effects of the experimental group on post-intervention outcomes after controlling for baseline scores were found for self-awareness (*F*(1, 140) = 18.19, *p* < 0.001, *ηp^2^* = 0.115), mindful attention (*F*(1, 140) = 16.24, *p* < 0.001, *ηp^2^* = 0.22), anxiety (*F*(1, 140) = 12.42, *p* < 0.001, *ηp^2^* = 0.08), stress (*F*(1, 140) = 23.11, *p* < 0.001, *ηp^2^* = 0.14), online vigilance (*F*(1, 140) = 18.66, *p* < 0.001, *ηp^2=^* 0.118), FoMO (*F*(1, 140) = 5.49, *p* < 0.001, *ηp^2^* = 0.04), deficient self-regulation (*F*(1, 140) = 6.60, *p* < 0.001, *ηp^2^* = 0.045), self-efficacy (*F*(1, 140) = 9.40, *p* < 0.001, *ηp^2^* = 0.063), impulsivity (*F*(1, 140) = 15.91, *p* < 0.001, *ηp^2^* = 0.10), problematic social media use (*F*(1, 140) = 6.96, *p* < 0.001, *ηp^2^* = 0.05), and smartphone use/day (*F*(1, 140) = 4.43, *p* < 0.001, *ηp^2^* = 0.03). No intervention effects were found for the intervention group for the variables of social media use/day (*F*(1, 140) = 3.697, *p* = 0.06), habit strength (*F*(1, 140) = 0.78, *p* = 0.78), and NoMO (*F*(1, 140) = 7.714, *p* = 0.91). ITT analyses demonstrated similar patterns to the PP samples’ outcomes.

### 3.3. Intervention Effects Based on Distraction Severity

In order to evaluate the effects of the intervention in the intervention group based on level of distraction and to assess whether the effects were consistent in the intervention group independent of degree of distraction, participants were classed into two categories of high distractors vs. low distractors depending on perceived distraction level. A median-split analysis with high vs. low distractor levels was determined by scores above vs. below the median and these were separately analysed inside the intervention group. Therefore, a two-way mixed ANOVA with time (pre-test and post-test) as within-factor and distraction severity (high and low distraction) as between-factor was performed to investigate the impact of the intervention (time) and degree of distraction (high vs. low) as assessed at baseline on distraction levels at post-intervention. This analysis was conducted only for the dependent variable for which the interactions were found to be significant.

Results indicated there was a significant main effect of the intervention *F*(1,70) = 77.17, *p* < 0.001. There was a significant main effect of distraction *F*(1,70) = 21.48, *p* < 0.001 with high distractors (*M* = 48.67) benefiting more than the low distractors (*M* = 33.54). Additionally, there was a significant interaction between the distraction status (high vs. low) and the degree of distraction *F*(1,70) = 20.10, *p* < 0.001. No significant interactions were found for self-awareness (*F*(1,70) = 1.07, *p* = 0.32); stress (*F*(1,70) = 0.17, *p* = 0.68); online vigilance (*F*(1,70) = 0.98, *p* = 0.32), deficient self-regulation (*F*(1,70) = 0.22, *p* = 0.64), self-efficacy (*F*(1,70) = 0.22, *p* = 0.64), anxiety (F(1,70) = 1.73, *p* = 0.19), and social media use (*F*(1,70) = 19.28, *p* = 0.30). However, significant main effects were also found for self-awareness (*F*(1,70) = 30.05, *p* < 0.001), deficient self-regulation *F*(1,70) = 20.10, *p* < 0.001, stress (*F*(1,70) = 47.95, *p* < 0.001), online vigilance *F*(1,70) = 42.07, *p* < 0.001, problematic social media use *F*(1,70) = 9.94, *p* < 0.05; FoMO (*F*(1,70) = 10.33, *p* < 0.001) and smartphone use/day (*F*(1,70) = 53.12, *p* < 0.001).

### 3.4. Mediation Analyses

More specifically for mediation 1, the intervention group was the proposed independent variable in these analyses, mindfulness was the proposed mediator, and smartphone distraction was the outcome variable. For mediation 2, stress was the proposed independent variable in these analyses, online vigilance was the proposed mediator, and smartphone distraction was the outcome variable. For mediation 3, smartphone distraction was the predictor, social media addiction was the outcome and online vigilance was the mediator. Analysed variables included the T1 scores on the constructs examined as covariates to account for pre-intervention performance.

For mediation 1, it was hypothesized that mindful attention would mediate the relationship between the intervention and smartphone distraction (Table 5). No mediation effect was found for mindful attention on the variables. However, a main effect of the intervention on smartphone distraction (path a: *b* = −0.67, *t* = −8.23, *p* < 0.001) was found, but no main effect of mindful attention on smartphone distraction (path b; *b* = 1.16, *t* = 0.67, *ns*).

For mediation 2, it was hypothesized that self-awareness would mediate the relationship between the intervention and smartphone distraction (Table 5). An indirect effect was found on self-awareness on the variables (a × b: *b* = −2.02, BCa CI = [−3.10, −1.59]), indicating mediation. The intervention significantly predicted self-awareness (path a; *b* = −6.78, *t* = −4.32, *p* < 0.001) and self-awareness significantly predicted lower levels of smartphone distraction (path b; *b* = 0.30, *t* = 4.02, *p* < 0.001).

For mediation 3, it was hypothesized that online vigilance would mediate the relationship between distraction and social media addiction (Table 5). An indirect effect was found on self-awareness on the variables (a × b: *b* = 0.02, BCa CI = [0.01, 0.03]), indicating mediation. The intervention significantly predicted self-awareness (path a; *b* = −0.01, *t* = −3.32, *p* < 0.001) and self-awareness significantly predicted lower levels of smartphone distraction (path b; *b* = 1.66, *t* = 4.02, *p* < 0.001).

## 4. Discussion

The present study tested the efficacy of an online intervention employing an integrative set of strategies—consisting of mindfulness, self-monitoring and mood tracking—in assisting young adults to decrease levels of smartphone distraction and improve on a variety of secondary psychological outcomes, such as mindful attention, emotional awareness, stress and anxiety, and perceived self-efficacy, as well as to reduce stress, anxiety, deficient self-regulation, problematic social media use and smartphone-related psychological outcomes (i.e., online vigilance, FoMO and NoMO). Results of the present study provided support for the online intervention effectiveness in impacting these outcomes. Findings suggested that students receiving the intervention reported a significant reduction in the primary outcome of smartphone distraction, unlike students in the control group who reported a non-significant reduction in smartphone distraction. In terms of the secondary outcomes, participants in the intervention condition experienced a significant increase in self-awareness, mindful attention, and self-efficacy, and a significant decrease in smartphone use/day, impulsivity, stress, anxiety, deficient self-regulation, FoMO, and problematic use. No significant results were found for social media use per day, habitual/automated use and NoMO.

According to the findings of the present intervention, it appears likely that practising mindfulness and monitoring mood and smartphone activity could lead to a desired behavioural change towards less distraction and less perceived stress with carry-over effects in self-awareness and self-efficacy, similar to interventions for other mental health problems [83,85,87,91,93,184,185]. These findings are consistent with the growing body of research indicating that mindfulness and self-monitoring are effective strategies to increase self-awareness and reduce stress [84,85,86,87,88,89,90,186]. Mindful attention could enhance awareness of individual media behaviour by: (i) raising understanding and awareness of disruptive media multitasking activities (i.e., predictors, patterns and effects), and (ii) raising awareness of different strategies for coping with digital distraction and of which strategies are most effective. Second, self-monitoring could help in developing an understanding of media habits and time spent on smartphone and social media activities and could curb perceived excess smartphone interaction, consistent with other study findings [92,101,187,188]. Therefore, strategies employing increased mindfulness practice and self-monitoring could aid attentional capacity and self-awareness, which is considered a necessary condition in the behaviour change process of risky behaviours [189,190].

Third, mood tracking could enhance awareness of triggers of negative mood and ensuing negative emotional states acting as drivers for distraction. It appears that the same technologies which may impact negatively on young people may be used to leverage smartphone use [100] and deflect psychological distress if evidence-based behaviour change strategies are applied. Intervention strategies such as mindfulness and self-monitoring may encourage increased self-awareness and thus help reduce distraction levels and increase mindful attention.

The intervention was also successful in reducing secondary outcomes, such as stress levels and FoMO, and it had a positive effect on emotion regulation and loss of control levels. Distraction appears to be associated with higher access to social media content and is mediated by online vigilance. Salience of smartphone-mediated social interactions (i.e., the salience dimension of online vigilance) has been found to be negatively related to affective wellbeing [49]. It has been reported that emotional dysregulation mediates the relationship between psychological distress and problematic smartphone use [191]. Higher self-regulation online has been identified as a moderator between need to belong and problematic social media use in young people [192] and emotion dysregulation as a mediator between insecure attachment and addiction [193]. Although distraction is an emotion regulation strategy with a protective function against emotionally distressing states [111] and dysphoric mood [194], or is used for adaptive coping [195,196], deficits in attentional control, such as distraction, may also be implicated in stress, anxiety or other affective disorders [197] and in generalized anxiety disorder with core cognitive symptoms related to excessive thoughts and deficits associated with increased perseverative worry [198]. Therefore, higher mindful attention and monitoring of mood may have influenced the reduction of distraction and the enhancement of emotional control.

Mediation analyses were also performed to understand the relationships between intervention effects on smartphone distraction via two mediators, mindful attention and self-awareness, and of online vigilance on the relationship between distraction and social media addiction. Mediation effects were significant for the relationship among intervention effects and distraction via self-awareness, and for distraction and problematic social media use via online vigilance, indicating that self-awareness could be a potential behaviour strategy to mitigate distraction levels. However, the relationship among intervention effects and distraction was not significant via mindful attention as a mediator. Therefore, in the present study it appeared that despite its statistically significant increase, mindful attention was not a mediating factor for distraction in the intervention. Mindful attention could potentially be the vehicle to increasing emotional self-awareness [93,184,199], prompting more controlled smartphone interactions. On the contrary, online vigilance was found to be a mechanism associated with smartphone distraction and problematic social media use, given the strong preoccupation with the content prompted even by the mere presence of smartphones, confirming previous findings [200].

Therefore, despite its protective function, distraction may concurrently serve as a gateway to increased smartphone engagement and time spent on devices. Time spent alone is not a defining factor and it has been argued instead that the interaction of content, context and time spent, as well as the meaning attached to these interactions, may determine the level of problematic media use [5,201]. Within smartphone use, distraction is a salient behaviour with evidence that distraction and mind-wandering are associated with online vigilance, which via reduced mindfulness may be associated with decreased wellbeing [78]. Furthermore, inattention symptoms have been implicated in risk for smartphone addiction and problematic smartphone use [202]. Therefore, handling distraction, which has neural correlates [203], may be the means to resisting cue reactivity, implicated in smartphone addiction, in reduced cognitive performance [113] or in obsessive-compulsive symptoms [204]. Further research is required to assess these cognitive and emotive dimensions of smartphone distraction and its effects on engagement in line with current trends [205]. However, it has been proposed that the construct of distraction extends beyond the debate on smartphone addiction by considering the role of the smartphone in coping with negative emotions and addressing preference for online vs. offline communications [206].

Research is still conflicted in relation to the cognitive function of distraction. Experimental smartphone research has provided initial evidence that social apps compared to non-social apps on smartphones do not capture attention despite their perceived high reward value [207,208], but other studies support a high interference effect [209]. Therefore, more research is required to elucidate the mechanisms of digital distraction and delineate how digital technologies, individual choices, and contexts affect individuals’ attention spans and attentional loss, as well as mental health conditions, such as ADHD and anxiety and overall psychological wellbeing [210]. The present RCT assessed the effectiveness of the impact of the use of mindfulness, self-monitoring, and mood tracking delivered through interaction with smartphone apps in reducing distraction arising from recreational smartphone use and social media use. The findings suggest that engaging with the aforementioned practices was effective in reducing distraction levels, stress, anxiety, deficient self-regulation, impulsivity and smartphone-related psychological outcomes, and improving mindful attention and emotional self-awareness and self-efficacy.

### Limitations, Implications, and Recommendations

Some limitations need to be taken into consideration. First, a convenience sample of university students was used, which hinders the generalizability of the findings to other groups (i.e., older adults or children). However, this population was considered of primary interest for the study because university students are digital natives liable to experience negative academic consequences due to vulnerability to problematic smartphone use [211].

The effect sizes found in this RCT were medium to large for the variables examined, exceeding the expected range for low-intensity, non-clinical interventions [212]. However, as a result of the main recruitment protocol, the intervention may have attracted participants who had an interest in the outcomes and a potential self-assessed vulnerability. Therefore, the voluntary, self-selected nature of participation could have introduced a significant degree of participant response and confirmation bias [213], resulting in the medium to high effect sizes. Additionally, the high drop-out rates, consistent with other online RCTs [214], could have significantly affected the strength of the findings [215], and the use of a passive control group might have led to an overestimation of the effects [216]. Due to the use of market-available apps, actual adherence and engagement with the intervention was not accounted for, nor were reasons for dropout [217]. Therefore, the findings should be treated with caution and replicated in future designs. Future studies should systematically address response bias and include methods in the RCT to improve the accuracy of self-reported data [218,219]. Combining self-report with behavioural data [220], ecological momentary sampling [221], psycho-informatics and digital phenotyping, the provision of a digital footprint for prognostic, diagnostic and intervention purposes [222], could enhance the ecological validity of the study. Equally, incorporating the measurement of brain activity using magnetic resonance imaging (MRI) in interventions could greatly enhance accuracy of assessment of prevention efforts and understanding of the role of neurobiology in behaviour [223,224].

The impact of the intervention on gender was not examined because this university student sample consisted mainly of female participants. Considering the gender differences reported in smartphone use [48,225] and in attention processes [226], future studies should explore its effect, which could have significant implications for the intervention and prevention of attention failures and poor student outcomes [227]. Additionally, the study design did not manage to provide a longer intervention period due to the lack of freely available apps for participants to use and did not include a second follow-up period to track maintenance of long-term effects, as is customary in RCTs, or the use of qualitative process evaluation for a critical understanding of impact of the intervention components [228]. Finally, social, economic and family conditions as well as other issues, which are critical to young people’s psycho-emotional states and sense of identity, were not accounted for in the present study [229,230].

Despite these limitations, the study provides initial evidence for efficacy of strategies in curbing smartphone distraction and adds to the limited body of knowledge of cognitive-emotive processes in smartphone and social media use [205]. It also contributed to the still limited knowledge on interventions in smartphone distraction and constitutes a simple, first-step, low key intervention programme, which may be practised by individuals seeking support for attentional difficulties on a self-help basis or within a stepped-care clinical framework for prevention purposes [96]. Experiencing distraction from smartphones and social media content, interferes with high-level cognitive processes and has productivity and emotional implications (i.e., stress) in various contexts and situations [51,231,232,233,234], being further compromised by digital triggers and the structural design of smartphones prompting salience and reactivity [235].

These results have clinical implications as low-intensity interventions may prevent small scale emotional problems from developing into clinical disorders and can reduce incidences of mental health problems [236,237]. Practitioners may also find value in using mindfulness and monitoring practices as an adjunct to therapy for problematic use of smartphones. It may be of high value for academic institutions to build specific university-based programmes on maintaining balanced technology use, tackling unregulated and promoting positive smartphone use, or guiding students towards suitable methods to address attention problems more effectively [238,239]. Apps may also be utilized by schools for students that are faced with attentional/excessive use difficulties and in assisting young people to become aware of their emotions in preparation for learning more adaptive coping strategies. Distraction is an emergent phenomenon in the digital era considering that the boundaries between work and recreation are increasingly blurred with both domains arguably dependent on the use of digital media [240]. More research on attentional processes within smartphone use could aid the understanding of these processes and impacts experienced across different age groups.

## 5. Conclusions

Psychological low-cost interventions may be effective in addressing precursors of problematic behaviours and enhancing wellbeing dimensions. The aim of the present study was to assess the efficacy of an RCT combining evidence-based cognitive-behavioural strategies to reduce distraction from smartphone use, increase mindful attention, emotional self-awareness and self-efficacy and reduce stress, anxiety, deficient self-regulation and smartphone related psychological outcomes (i.e., online vigilance, FoMO and NoMO). Second, it tested the mediating effect of mindful attention and self-awareness of the intervention on distraction, and of online vigilance on the relationship between distraction and social media addiction.

Findings suggested that students receiving the intervention reported a significant reduction in the primary outcome of smartphone distraction, whereas students in the control group reported a non-significant reduction in smartphone distraction. In terms of the secondary outcomes, participants in the intervention condition experienced a significant increase in self-awareness, mindful attention and self-efficacy and a significant decrease in smartphone use/day, impulsivity, stress and anxiety levels, FoMO, deficient self-regulation and problematic social media use. No significant results were found for duration of social media use/day, habitual use and NoMO. Mediation effects of the intervention were also observed on distraction and problematic social media use via the mediators of emotional self-awareness and online vigilance in mitigating distraction levels. Mindful attention was not found to be a mediating process for reducing distraction in the intervention.

Research on digital distraction is still scarce, yet there is increasing interest in cognitive impacts within digital environments. More evidence is required to assess the nature of attention failures and difficulties occurring both in normative and excessive online use. This evidence would allow an understanding of the prevalence and the nature of these difficulties, as well as their integration in intervention media literacy and risk prevention programmes, enhancing wellbeing, productivity and academic performance.

## Figures and Tables

**Table 1:** The three components of the intervention.

Intervention Components	Smartphone App Used	Evidence-Based Benefits	Psychological Evidence for Benefits
Mindfulness			
Brief mindfulness sessions	Headspace app	Mindfulness practice and mood tracking offer benefits in emotion regulation, attention, stress and low mood levels & meta-awareness Evidence for replenishing students’ focused engagement in mental tasks (i.e., homework)	[128] [80] [82,83] [129]
Self-monitoring and Self-exclusion			
Social media and smartphone use Abstinence option	Anti-Social app	Self-monitoring & exclusion (minutes on social media, times of unlocking smart phone each day, favourite and most time consuming and accessed apps) aid emotion regulation Reflection on dependence on smartphone, extent of use, lost attention, checking frequency Performance feedback & meta-awareness	[130] [131] [77] [132] [133] [134] [101]
Mood-tracking			
	Pacifica app	Mood tracking can boost overall emotional self-awareness which can in turn lead to improvements in emotional self-regulation	[93] [135] [136]

Daily reminders and messages via blogging were sent as a reminder to maintain routine and reflect on levels of activity [126,137].

**Table 2:** Per protocol baseline sociodemographic, usage data, psychological variables and pre-post intervention scale reliabilities.

	Intervention (*n* = 72)		Control (*n* = 71)		Chi Square/ *t*-Tests		
Socio/demographics	*n*	%	*n*	%		-	-
Gender (female)	60	83.33	62	87.32	1.83, *ns* ^a^		
Education (under graduates %)	67	93.05	65	91.54	1.03, *ns*		
Relationship status (% not in relation)	40	55.55	38	53.52	1.35, *ns*		
Ethnicity (White %)	49	68.05	42	59.15	1.63, *ns*		
	***M (SD)***		***M (SD)***		***t* Tests**	**Cronbach’s** **α T1**	**Cronbach’s α T2**
Age	20.69 (3.27)		20.82 (3.70)		−0.20, *ns*	-	-
Smart hours/day	4.55 (2.28)		5.23 (1.89)		−0.28, *ns*	-	-
SM hours/day	2.17 (1.430		2.47 (1.28)		−1.36, *ns*	-	-
Smart. distraction	59.52 (7.69)		57.55 (8.08)		−0.70, *ns*	0.90	0.88
Self-awareness	74.71(8.20)		75.00 (9.38)		−0.20, *ns*	0.87	0.86
Mindful Attention	3.28 (0.52)		3.40 (0.56)		−1.32, *ns*	0.92	0.93
Stress	24.44 (4.72)		28.78 (6.05)		−0.33, *ns*	0.86	0.83
Anxiety	15.93 (5.94)		16.63 (4.94)		−0.77, *ns*	0.93	0.90
Online vigilance	2.43 (0.48)		2.38 (0.52)		0.63, *ns*	0.89	0.87
Efficacy	28.04 (4.35)		28.96 (4.55)		−2.51, *ns*	0.90	0.88
FoMO	3.48 (1.36)		3.54 (1.34)		−0.32, *ns*	0.89	0.90
NoMO	77.17 (22.40)		86.32 (23.68)		−0.49., *ns*	0.89	0.88
Def. self-regulation	14.15 (5.32)		15.35 (5.39)		−1.50, *ns*	0.89	0.87
Impulsivity	14.74 (3.39)		16.27 (3.52)		−0.264, *ns*	0.85	0.86
Prob. SM use	17.15 (4.95)		17.18 (5.42)		−0.035, *ns*	0.91	0.89
Automaticity	5.14 (1.33)		5.11 (1.20)		−0.88, *ns*	0.87	0.89

a ns = non-significant. FoMO=Fear of Missing Out; NoMO = Nomophobia; Def. self-regulation=Deficient self-regulation; Prob. SM use = Problematic social media use.

**Table 3:** Bivariate Pearson’s *r* correlation analyses.

	1	2	3	4	5	6	7	8	9	10	11	12	13	14
1. Distraction	1													
2. Stress	0.199 **	1												
3. Pr. SM use	0.631 **	0.173 **	1											
4. Mind.Att.	−0.523 **	−0.145 *	−0.455 **	1										
5. Self-Aware	−0.340 **	0.057	−0.318 **	−0.209 **	1									
6. Anxiety	0.460 **	0.380 **	0.435 **	0.450 **	0.242 **	1								
7. Onl. Vigil.	0.507 **	0.280 **	0.620 **	0.380 **	0.223 **	0.283 **	1							
8. Efficacy	−0.107	−0.343 **	−0.149 *	−0.101	0.148 *	−0.399 **	−0.056	1						
9. Automat	0.575 **	0.286 **	0.466 *	0.324 **	0.194 **	0.304 **	0.348 **	−0.179 **	1					
10. Impuls.	0.455 **	0.006	−0.053	−0.037	−0.522	−0.026	0.035	0.086	0.037	1				
11. Def. Self-reg.	0.333 **	0.048	0.017	0.048	−0.068	0.007	0.074	0.025	0.049	0.859 **	1			
12. Smart/day	0.314 **	−0.280	0.013	−0.128	−0.025	−0.161	0.082	0.021	−0.145	−0.008	−0.004	1		
13. SM/day	0.116	0.004	−0.025	−0.008	−0.109	0.024	−0.035	−0.111	0.061	0.154	0.168 *	0.423 **	1	
14. FoMO	0.281 **	0.323 **	0.382 **	0.103	0.310 **	0.369 **	−0.032	−0.164 **	0.235 **	0.026	0.035	0.183 **	0.180 **	1
15. NoMO	0.513 **	0.375 **	0.421 **	0.007	0.142 *	0.312 **	0.136 *	−0.209 **	0.392 **	−0.084	−0.084	0.189 **	0.096	0.341 **

* *p* < 0.05; ** *p* < 0.01; *** *p* < 0.001. Pr. SM use: Problematic social media use; Mind. Att: Mindful attention; Onl. Vigil.: Online vigilance; FoMO: Fear of Missing Out; NoMO: Nomophobia; Def. self-regulation: Deficient self-regulation; SM/day; Social Media use/day.

**Table 4:** Per protocol sample (*n* = 143) primary and secondary measures, means, SDs, effect sizes and *F*-values for between-group comparisons.

Measure	Experimental (*n* = 72)		Control (*n* = 71)		Effect	Effect Size	Cohen’s *d*
	Pre	Post	Pre	Post	*F*	*ηp2*	*d*
	*M(SD)*	*M(SD)*	*M(SD)*	*M(SD)*			
Smart.Distraction	58.06 (7.69)	39.70 (17.67)	59.72 (8.08)	58.78 (17.47)	46.59 ***	0.25	1.11
Self-awareness	74.71 (8.20)	83.30 (9.89)	75.00 (9.38)	76.25 (10.25)	18.19 ***	0.12	0.69
Mind.Attention	3.28 (0.52)	3.97 (0.69)	3.40 (0.56)	3.37 (0.76)	16.24 ***	0.22	0.82
Stress	24.44 (4.72)	24.10 (4.63)	28.78 (6.05)	27.94 (5.24)	23.11 ***	0.14	0.77
Anxiety	15.93 (5.94)	14.75 (4.43)	16.63 (4.95)	17.44 (4.42)	12.42 ***	0.08	0.60
Vigilance	2.43 (0.49)	1.98 (0.63)	2.38 (0.52)	2.39 (0.52)	18.66 ***	0.12	0.70
Self-efficacy	28.04 (4.36)	32.32 (5.08)	28.96 (4.55)	29.99 (5.05)	9.40 ***	0.06	0.46
FoMO	3.48 (1.36)	2.86 (1.16)	3.54 (1.34)	3.32 (1.22)	5.49 ***	0.04	0.39
NoMO	77.17 (2.40)	78.03 (2.72)	86.32 (23.6)	79.50 (2.74)	7.71	-	-
Def. self-reg.	17.16 (6.70)	14.00 (5.32)	17.61 (6.91)	15.32 (5.39)	6.60 ***	0.04	0.25
Impulsivity	17.32 (3.79)	14.74 (3.41)	17.65 (3.92)	16.27 (3.51)	15.91 ***	0.10	0.44
Probl. SM use	17.15 (4.95)	15.12 (4.40)	17.18 (5.42)	17.24 (5.11)	6.96 ***	0.05	0.44
Automaticity	5.14 (1.33)	4.77 (1.30)	5.11 (1.20)	4.98 (1.59)	0.78	-	-
SM. use/day	2.92 (1.75)	2.17 (1.44)	2.89 (1.52)	2.47 (1.28)	3.70	-	-
Smart. use/day	4.51 (2.28)	3.51 (1.88)	4.45 (1.89)	4.11 (1.68)	4.43 ***	0.03	0.34

* *p* < 0.05; ** *p* < 0.01; *** *p* < 0.001.

**Table 5:** Mediation effects of mindful attention and emotional self-awareness on intervention effects and smartphone distraction and of online vigilance on smartphone distraction and social media addiction (*n* = 252).

Predictor	Outcome	Mediator	*ab (B)*	*a*	*b*	*c*	*c′*
Intervention	Smart.Distract.	Mindful Att.	−0.79 [−3.10, −1.59]	−0.67 [−0.84, −0.51]	1.16 [−2.25, 4.58]	20.75 [16.35, 25.16]	21.55 [16.62,26.48]
Intervention	Smart.Distract.	Self-aware	−2.02 [−3.97, −0.35]	−6.78 [−9.15, −4.40]	0.30 [0.07, 0.52]	20.91 [16.59, 25.22]	22.93 [18.38, 27.48]
Smart. distract.	Probl. SM use	On.vigilance	0.02 [0.01, 0.03]	0.01 [0.010, 0.015]	1.66 [0.78, 2.54]	0.11 [0.08, 0.13]	0.089 [0.06, 0.12]
